# Urinay neutrophil gelatinase-associated lipocalin as a biomarker in different renal problems

**DOI:** 10.3906/sag-2002-130

**Published:** 2020-10-22

**Authors:** Didem TURGUT, Serhan Vahit PİŞKİNPAŞA, Ezgi COŞKUN YENİGÜN, Nihal AYDEMİR, Fatih DEDE

**Affiliations:** 1 Division of Nephrology, Department of Internal Medicine, Başkent University Ankara Hospital, Ankara Turkey; 2 Department of Nephrology, İskenderun State Hospital, İskenderun Turkey; 3 Department of Nephrology, Ankara Numune Education and Research Hospital, Ankara Turkey; 4 Division of Nephrology, Department of Internal Medicine, Faculty of Medicine, Hitit University, Çorum Turkey

**Keywords:** Acute kidney injury, urinary neutrophil gelatinase-associated lipocalin (uNGAL), postrenal, cut-off value

## Abstract

**Background/aim:**

Neutrophil gelatinase-associated lipocalin (NGAL) is used previously to estimate the etiology, severity, and clinical outcomes of acute kidney injury (AKI). However, the role of urinary NGAL (uNGAL) in the postrenal setting is not clear. In our study, we aimed to discover the cut-off value of uNGAL that can be used in the differential diagnosis of underlying AKI etiologies.

**Materials and methods:**

In this prospective cross-sectional study, we examined 82 subjects in four groups: patients that had (1) postrenal AKI; (2) AKI other than postrenal etiologies; (3) stable chronic kidney disease; and (4) healthy subjects. A renal function assessment was carried out by measuring serum creatinine (sCr) and uNGAL at the time of diagnosis [0th min (T0)]. We followed the study group for three months.

**Results:**

At the time of diagnosis, sCr (T0) was highest in the postrenal AKI and AKI groups in contrast to stable chronic kidney disease patients and healthy subjects (P < 0.001), as expected. T0 median uNGAL was highest in the postrenal group (P < 0.001). Area under curve (AUC) of uNGAL to estimate postrenal AKI presence was 0.957 (95% CI, 0.897–1.000; P < 0.001). The cut-off point of uNGAL was 42.625 ng/mL for this estimation.

**Conclusion:**

Patients with AKI must be classified according to the underlying etiologies as soon as possible. uNGAL may be useful to estimate the etiologies, and whether the problem is acute or chronic in the course. In postrenal kidney problems, to plan the urgency of the urologic procedures, it is crucial.

## 1. Introduction

In some renal function problems, clinicians may not understand whether a patient had acute, subacute, or chronic kidney injury [1–3]. Knowing the duration and severity of the problem’s onset narrows the spectrum of the patients who may benefit from early interventions. Besides, it prevents clinicians from unnecessary workups. Contrary to popular myth, sCr, serum urea, and urine output are suboptimal functional markers to estimate the onset and type of kidney injury. However, uNGAL can predict acute kidney impairment in the early phase, and can assess etiology, severity, or clinical outcomes of AKI [4–8]. uNGAL tested in multiple studies of patients with AKI, but its role in postrenal causes is not clear. In some postrenal AKI conditions, it is not apparent whether the obstruction is chronic or acute, if it is not total. And the precise timing of removal of the obstruction is not visible. In this study, we aimed to determine the accuracy of uNGAL as a marker of underlying AKI etiologies who admitted to the hospital. We objected to estimating a cut-off value for uNGAL to understand whether kidney problem is acute or not, especially in a postrenal setting.

## 2. Materials and methods

### 2.1. Study design 

We performed a prospective study with patients admitted to Ankara Numune Education and Research Hospital and referred to the department of nephrology during the three months. We enrolled patients in four groups: (1) patients with postrenal AKI (pAKI); (2) patients with AKI other than postrenal causes (AKI); (3) patients with stable CKD (sCKD); and (4) healthy control group. The glomerular filtration rate (GFR) was estimated using the modification of diet in renal disease equation (MDRD) [9]. AKI was defined according to several parameters:

1. New-onset as an increase in sCr by ≥ 0.3 mg/dL within 48 h,

2. Increase in sCr to ≥ 1.5 times baseline (which is known or presumed to have occurred within the prior seven days),

3. Urine volume < 0.5 mL/kg/h for 6 h [10].

Patients without known baseline sCr were not classified as having AKI. All patients with postrenal AKI and AKI were in stage 3 AKI [11]. sCKD was defined as a reduced estimated GFR of <60 mL/min/1.73 m2 before admission (>3 months) and no change from baseline during the hospitalization period [12]. The Charlson comorbidity index (CCI) was used to measure patients’ comorbidities [13]. Exclusion criteria for the general study population were age <18 or > 75 years old, pregnancy, and/or any infectious condition. 

### 2.2. Patient population

**pAKI group: **Seventeen patients enrolled in the group. Patients were selected due to AKI criteria and ultrasound (USG) findings. All patients had bilateral grade 2–3 hydroureteronephrosis on the USG, and none of them were anuric. After a urethral catheter inserted into each patient, all patients were evaluated with an urologist concerning for percutaneous nephrostomy need. Interventional radiologists placed a percutaneous nephrostomy tube into each of the 17 patients at the end of the evaluation. Using the Seldinger technique, a tube ranging from 8–12F was placed with USG. Placement of the nephrostomy catheter time was considered as the 0th-min (T0), and blood samples (sCr) and urine specimens for NGAL analysis were collected at the same time as 0th min evaluation (T0 sCr). Patients with unilateral ureteral dilation or with known vesicoureteral reflux were excluded.

**AKI group: **Twenty patients were enrolled in this group. Patients were selected based on AKI criteria. AKI was defined as a new-onset increase in sCr [10]. The examination time by a nephrologist was accepted as T0 time, and blood samples (T0 sCr) and voided urine specimen for NGAL (T0 uNGAL) analysis were collected. 

**CKD group: **Twenty-two patients were enrolled in this group. All patients were had stable sCr for >3 months and had been admitted to hospital for reasons other than kidney problems. Patients presented with stage 3–4 CKD. The nephrologist’s evaluation was accepted as T0 time, and blood samples (T0 sCr) and voided urine specimens for NGAL (T0 uNGAL) analysis were collected.

**Control group:** A group of 23 healthy subjects between 18 and 75 years old were prescreened by history and laboratory tests (blood, urine, and renal USG assessment) to exclude any preexisting urological or nephrological issues. They were recruited to provide voided bladder urine specimens for NGAL analysis. 

### 2.3. Laboratory measurements

Baseline renal function by SCr, estimated glomerular filtration rate (eGFR), medical history, and demographic characteristics were obtained from electronic hospital records. A prospective renal function assessment was carried out by measuring sCr at 0 and 48 h, and at 1 and 3 months later (T0, T48, T1, and T3, respectively) from admission. uNGALwas measured at T0 (as mentioned above) from all study groups. Urinary samples for NGAL measurements were collected in ethylene diamine tetra cetic acid (EDTA) tubes and centrifuged at 3000 × g for 5 min. Supernatants were quickly frozen at −80 °C. uNGAL was measured using Human Lipocalin-2/NGAL ELISA kits (Biovendor GmbH, Heidelberg, Germany, RD191102200R) following the manufacturer’s instructions exactly. All resulting values in the samples were in the range of the standard curve provided by the manufacturer.

### 2.4. Statistical analysis

Data analyses were performed using the computer program SPSS 15.0 (SPSS Inc., Chicago, IL, USA). Statistical analysis was performed using the one-way ANOVA for parametric data, and Kruskal–Wallis for nonparametric data. When the null hypothesis for these tests rejected, posthoc tests were used to find the differences. As posthoc tests, Tukey–Kramer, and six pairwise comparisons with Bonferroni correction were used. Differences between the groups were analyzed by Friedman’s analysis of variance (ANOVA) for repeated measures. The receiver operating characteristic (ROC) curve was used to determine an uNGAL level that estimates AKI etiology. We assessed ROC curves and calculated the area under the curve (AUC), including 95% confidence intervals (CI). Estimated cut-off values were determined with the Youden’s index. Univariate linear regression models were used to estimate uNGAL correlations with the first and third-month serum creatinine levels after the AKI period. A value of P = 0.05 was considered significant.

## 3. Results

### 3.1. Patient characteristics

**Figure 1:**
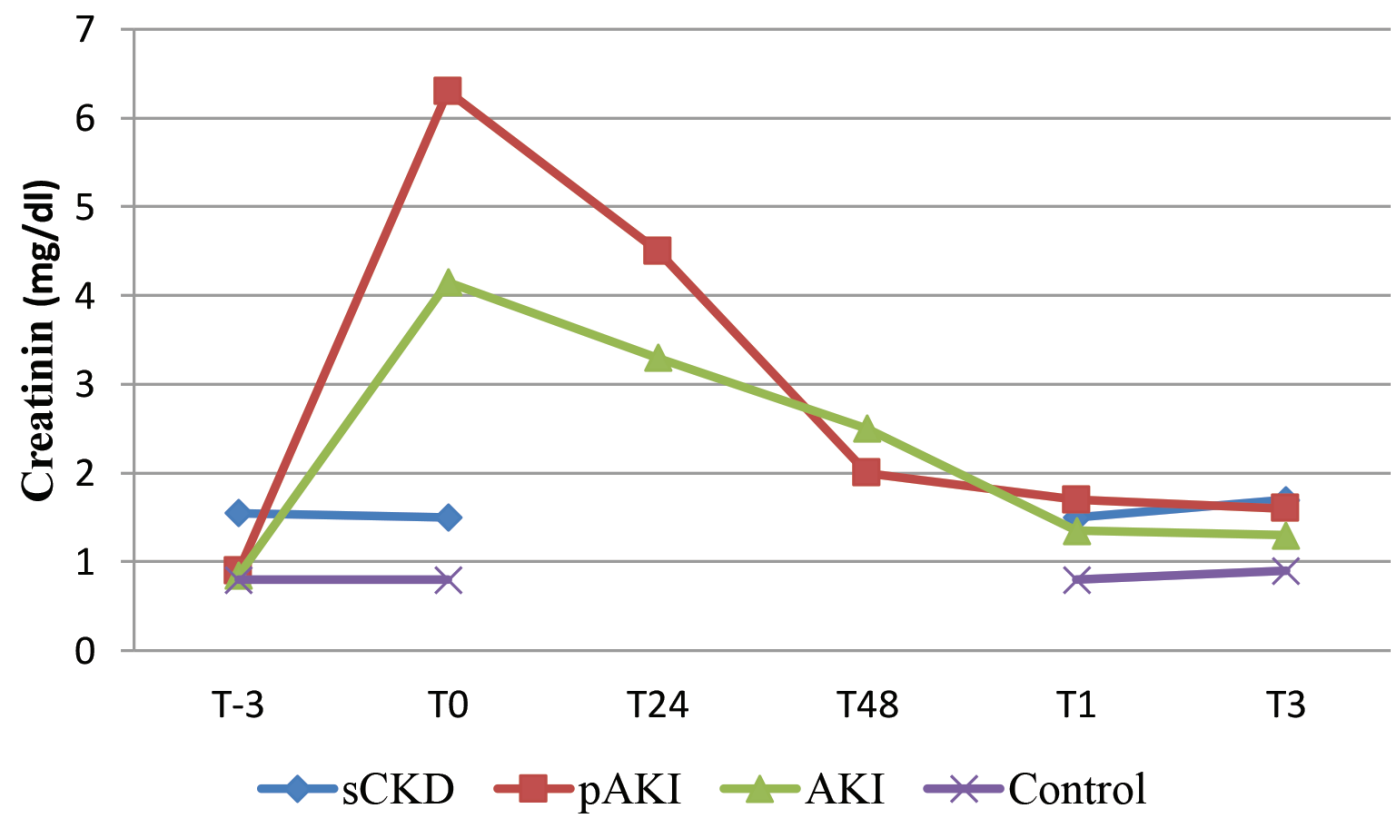
Serum creatinin changes during follow-up.

A total of 82 subjects were enrolled in the study. Seventeen patients with pAKI, 20 patients with AKI, 22 patients with sCKD, and 23 healthy subjects were analyzed. Six patients had hemodialysis (HD) in the pAKI group, and three patients had HD in the AKI group. None of the patients had renal replacement treatment (RRT) by the one- and/or three-month follow-up examination. Median CCI was higher in the pAKI group with respect to AKI and sCKD patients (P < 0.001). T0 sCr was higher in pAKI, AKI, and sCKD patients, but there was no statistical difference between the pAKI and AKI groups (Table 1). sCr changes during follow-up are summarized in Figure 1. The urinary tract obstructions in pAKIweredue to several reasons: (1) 7/17 cases of bladder cancer (41.1%); (2) 4/17 cases of prostate cancer (23.5%); and (3) 6/17 miscellaneous causes of obstruction (35.4%) as retroperitoneal fibrosis and post radiation. The AKI group was composed of prerenal or intrinsic etiologies without any surgical history. 

**Table 1:** Patient characteristics and laboratory findings.

	pAKI(n = 17)	AKI(n = 20)	sCKD(n = 22)	Control(n = 23)	P
Age, years	64.6 ± 10.5	60.3 ± 18.9	63.7 ± 11.2	32.9 ± 9.9	NS
Women, n (%)	6 (35.3%)	9 (45.0%)	12 (54.5%)	11 (47.8%)	NS
CCI (min-max)	6 (0–10)	2 (0–10)	5 (2–8)	0	
Hemodialysis, n (%)	6	3	0	0	
Laboratory values (min-max)					
T0 serum cr (mg/dL)	6.30 (2.40–11.30)	5.15 (2.20–13.20)	1.50 (1.00–3.40)	0.80 (0.60–1.00)	<0.001a
T48 serum cr (mg/dL)	2.00 (1.40–6.50)	2.50 (1.10–10.80)			0.456
T1 serum cr (mg/dL)	1.70 (0.80–4.20)	1.35 (0.60–11.40)	1.50 (1.10–3.00)	0.80 (0.50–1.00)	NS
T3 serum cr (mg/dL)	1.60 (0.50–4.30)	1.30 (0.50–7.40)	1.70 (1.00–3.10)	0.90 (0.08–1.00)	NS
T0 uNGALng/mL (min-max)	98.7 (32.2–137)	57.8 (5.2–121.0)	31.9 (23.4–47.2)	13.7(1.2–42.1)	<0.001b

### 3.2. Discriminative capability of uNGAL and cut-offs for renal function problems

T0 median uNGAL was highest in the pAKI group. When we compare the groups for T0 uNGAL, there was a statistical difference between the pAKI and control group (98.7 and 13.7, respectively; P < 0.001), pAKI and CKD groups (98.7 and 31.9, respectively; P < 0.001), pAKI and AKI groups (98.7 and 57.8, respectively; P = 0.0016), and AKI and control groups (57.8 and 13.7, respectively; P < 0.001). The differences, however, between the sCKD and control groups (31.9 and 13.7, respectively; P = 0.212) and AKI and sCKD groups (57.8 and 31.9, respectively; P = 0.578) were not statistically significant (Table 1).

**Figure 2:**
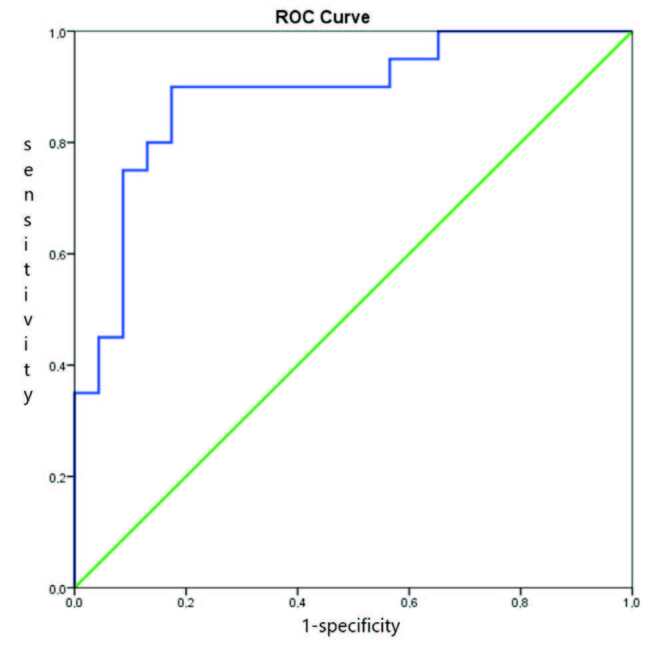
The ROC curve analysis of uNGAL for estimating AKI.

**Figure 3:**
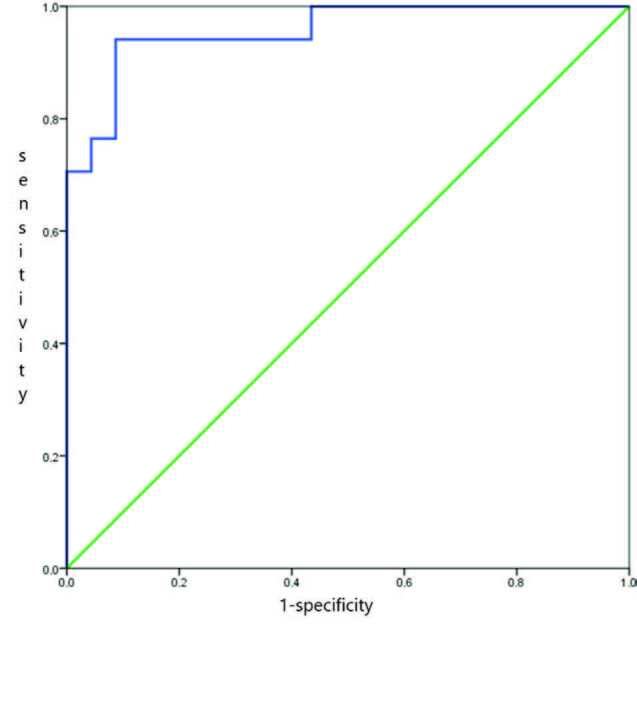
The ROC curve analysis of uNGAL for estimating postrenal AKI.

**Figure 4:**
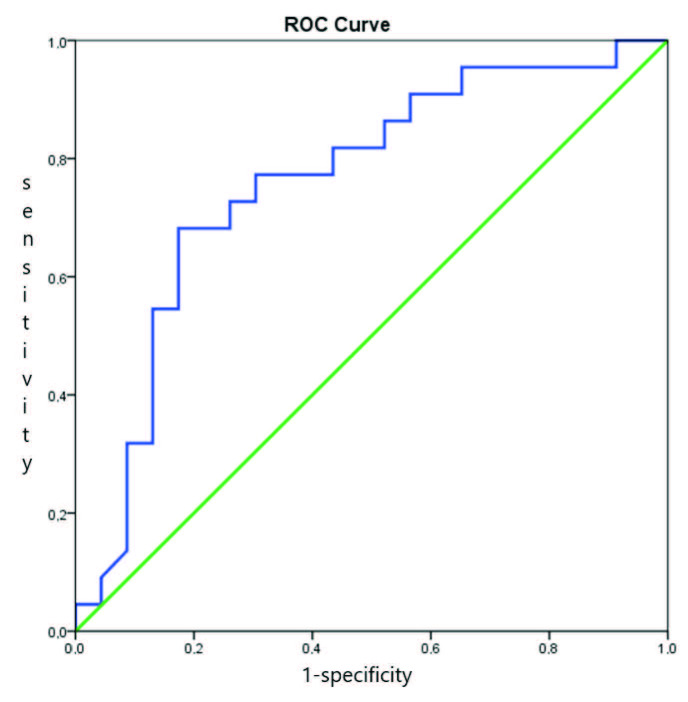
The ROC curve analysis of uNGAL for estimating sCKD.

In the ROC curve analysis, the AUC of uNGAL for estimating pAKI was 0.957 (95% CI, 0.897–1.000; P < 0.001). The cut-off point was 42.625, and the sensitivity and specificity were 0.94% and 0.91%, respectively, according to Youden’s index. The AUC of uNGAL for estimating AKI was 0.885 (95% CI, 0.781–0.988; P < 0.001). According to Youden’s index, the cut-off point was 26.205, and the sensitivity and specificity were 0.90% and 0.82%, respectively (Table 2). The ROC curves are presented as Figures 2–4.

We created simple linear regression models for uNGAL to predict sCr after one month of the AKI period (T1) and sCr after the third month of the AKI period (T3). Although there was statistically significant correlation, R-squared (R2) was 10.5% for uNGAL as an independent predictor for T1 (P < 0.001) and 16.9% for T3 (P < 0.001). 

**Table 2:** AUC for uNGAL estimating the type of kidney problem.

	ROC-AUC (SE)	95% CI	P	Cut-offng/mL	Sensitivity%	Specificity%
pAKI	0.957 (0.031)	0.897–1.000	<0.001	42.625	0.94	0.91
AKI	0.885 (0.053)	0.781–0.988	<0.001	26.205	0.90	0.82
sCKD	0.760 (0.074)	0.615–0.905	0.003	25.180	0.68	0.82

## 4. Discussion

Discrimination of underlying etiologies of AKI remains challenging in patients with multiple comorbidities and those who are critically ill. In this study, we investigated whether uNGAL has an additive value to differentiate sCr elevations concerning AKI etiologies. uNGAL has been studied extensively in different AKI models [12,14–16]. Singer et al. studied uNGAL for the diagnosis of intrinsic and prerenal AKI, and they found that an uNGAL level >104 µg/L indicated intrinsic AKI (likelihood ratio 5.97). In the same study, it was also presented that uNGAL independently predicted the composite outcome after correcting for demographics, comorbidities, creatinine, and RIFLE (risk, injury, failure, loss of kidney function, end-stage disease) class [17]. In postrenal settings, studies related to uNGAL are limited. Urbschat et al. investigated uNGAL and kidney injury molecule-1 (KIM-1) in acute postrenal impairment. In this study, 53 patients with a proven diagnosis of acute unilateral renal colic and 52 control patients were included. Mean sCr levels of patients were higher compared to the control group (1.11 mg/dL versus 0.92 mg/dL; P < 0.001). Serum NGAL (P < 0.001) and uNGAL (P < 0.001) were both increased in the obstructive nephropathy group [18]. In this study, although ureteral obstructions were not total or bilateral, the increase in NGAL levels was a good biomarker for the kidney problem rather than the increase in sCr. There are some studies involving pediatric patients; that all demonstrated that uNGAL level was found to be increased in congenital hydronephrosis [19,20]. 

To our knowledge, our study is the first one that used uNGAL as a urinary biomarker of AKI in different etiologies, concomitantly. Additionally, we studied the uNGAL levels in the setting of an acute bilateral urinary obstruction to discriminate the direct effect of obstruction on uNGAL levels. In the study, uNGAL was highest in patients with bilateral urinary obstruction (pAKI) compared to patients with prerenal-intrinsic AKI or sCKD. The difference in uNGAL between pAKI-AKI groups was not related to the severity of the AKI stage since all patients in the two groups were with stage 3 AKI. It is likely to speculate that an increase in uNGAL is related to mainly tubular function problems rather than a parenchymal injury or a chronic course. In an experimental model of unilateral ureteral obstruction study, authors showed histologically that NGAL protein synthesis significantly increased in the dilated thick ascending limb of Henle. NGAL found in the urine was retained considerably in the swollen pelvis of the ligated kidney [21], demonstrating the role of NGAL in postrenal AKI. This finding suggests that NGAL was more strongly synthesized in damaged epithelia. Our results correlated with this knowledge in which a significant increased uNGAL was associated with obstruction rather than intrinsic or prerenal conditions.

In the studies related to cut-off levels of uNGAL to predict severity, there are heterogeneous results. In our research, the uNGAL cut-off value was highest in the postrenal group (42.625 ng/mL). Wasilewska et al. presented a study, including children with obstructive nephropathy. They found that increase in uNGAL levels during the AKI course was associated with worsening obstruction, and it was independent of an increase in creatinine [19]. Since our entire pAKI patient group had a total bilateral urinary obstruction, it is likely that as uNGAL increases in the postrenal course, obstruction gets severe needing immediate urologic intervention. Our presented cut-off levels were lower than in other studies [4, 22,23], which would be related to a higher variability in the assay types used. In correlation analysis, we aimed to find whether spot uNGAL levels might show the renal prognosis after three months. We showed a significant but weak correlation between uNGAL levels and follow-up sCr levels (R2 10.5% and 16.9%, for the first and the third month, respectively) in the all study group. uNGAL should be analyzed with more patients to assess as a predictive marker in the postrenal setting.

This study has some limitations, like being a single-centered clinical trial study with a small sample size without additional follow-up of changes in uNGAL levels. Kostic et al. revealed that urinary biomarkers, alone and in combination, showed potential and noninvasive diagnostic tool for identifying infants with different levels of urinary obstruction who may benefit from earlier surgical intervention [24]. In our study, we did not analyze patients based on uNGAL levels after obstruction relief. Therefore, a large-scale, multicenter study is necessary to follow up on the biomarker level changes during the progress of obstructive nephropathy and confirm the value of uNGAL in renal prognosis monitoring. 

In conclusion, patients with pre and postrenal AKI must be identified as soon as possible while their treatments are completely different. Missing information about the baseline sCr level often confounds the interpretation of a current elevated sCr concentration if obstruction is mild or not total. uNGAL as an AKI biomarker can be useful for facilitating a differential diagnosis of chronic conditions and intrinsic, prerenal, or postrenal etiologies at an early point. Patients with pAKI (unilateral and/or subtotal) may greatly benefit from early interventions that resolve obstruction before a severe AKI stage established. Future studies with high patient numbers will guide nephrologists and urologists in this setting. 

**Conflict of interest**

The authors declare that they have no conflict of interest.

**Informed consent**

The study protocol was approved by the institution’s ethics committee (No. 2011-285) and was carried out under the Declaration of Helsinki. A signed informed parental consent form was obtained from all participants.
